# Is it a Drug or a Bug? A Case of Chemotherapy and Immune Modulators Complicating the Diagnosis of Ehrlichiosis

**Published:** 2018-08-30

**Authors:** Mason E. Uvodich, Victoria Poplin, Heather J. Male

**Affiliations:** 1 University of Kansas School of Medicine, Kansas City, KS; 2 Department of Internal Medicine; 3 Division of Hematologic Malignancies and Cellular Therapeutics (HMCT)

**Keywords:** ehrlichiosis, immunocompromised patient, leukemia, chemotherapy, tick-borne diseases

## INTRODUCTION

Ehrlichiosis is caused primarily by *Ehrlichia chaffeensis* and *Ehrlichia ewingii*.^1^,^2^ These obligate intracellular gram-negative cocco-bacilli are transmitted by arthropod vectors. They reside in vertebrate reservoirs and undergo a tick-mammal-tick cycle during which humans are dead-end hosts. The life cycle of *E. chaffeensis* is perpetuated predominantly via the *A. americanum* (Lone star) tick.^1^,^2^ During human infection, *E. chaffeensis* preferentially targets monocytes. Men are affected more often than women. Individuals between the ages of 65 and 69 had the highest incidence rate (IR) between 2008 and 2012.^1^,^3^ During this period, Oklahoma and Missouri had the highest IR at 30.9 and 26.3 per million, respectively.^3^ Patients with hematologic malignancies undergoing chemotherapy can have similar symptoms to those found in ehrlichiosis without being infected. However, in the appropriate setting, tick-borne illness should be considered. We present a case of severe illness and prolonged fevers due to ehrlichiosis in a patient who received chemotherapy for chronic lymphocytic leukemia (CLL).

## CASE REPORT

A 77-year-old man with CLL who completed six cycles of bendamustine and rituximab three months prior, presented in early August with complaints of fevers, night sweats, decreased appetite, and nausea for one month and was found to have a white blood count (WBC) of 1.5K/μL. This patient had asymptomatic persistent leukopenia following therapy into mid-May and mid-July. The patient was seen in the emergency department and in clinic by his oncologist, where he was febrile up to 39.3°C, and subjectively had fatigue, myalgias, and arthralgias. The work-up, including chest x-ray, blood cultures, and urinalysis, was negative. He was treated empirically with levofloxacin and amoxicillin-clavulanic acid. In addition, he received seven doses of filgrastim (human-granulocyte colony stimulating factor) during his course of antibiotics.

After completion of antibiotics, the patient had a brief period of a few days where he felt well enough to proceed with vacation plans. During his vacation, his symptoms recurred and worsened quickly. He presented to clinic immediately after his vacation and noted darkening of his urine secondary to presumed dehydration, in addition to his previous symptoms. At that time, his labs were significant for hyponatremia, elevated glucose, lactate dehydrogenase of 275 μ/L, AST of 56 μ/L, ALT of 56 μ/L, alkaline phosphatase of 91 μ/L, a normal WBC, and a platelet count of 271 x 106 /μL. Attempts were made to manage at home, but with ongoing worsening of his condition after three days, he was instructed by his oncologist to be admitted to the hospital.

New symptoms on admission included altered mental status and insomnia. On admission, he was febrile and physical exam was pertinent for rales in bilateral lung bases, and he was without rash, focal neurological deficits, or lymphadenopathy. CT of his sinuses, chest, abdomen, and pelvis demonstrated no abnormal lymphadenopathy or nidus of infection. His labs were notable for normal white blood count of 5.2 K/μL, with a platelet count of 49,000 K/μL. Ferritin was > 7500 ng/mL and his AST and ALT were 141 μ/L and 81 μ/L, respectively.

Based on his ferritin level, there was concern for hemophagocytic lymphohistiocytosis. A bone marrow biopsy demonstrated residual CLL without evidence of hemophagocytosis or macrophage predominance. On the second day of admission, infectious disease was consulted. They empirically started amphotericin, imipenem, and doxycycline. Over the next 24 hours, the patient worsened with persistent fever, rigors, altered mental status, tachypnea, and a new oxygen requirement. Repeat chest imaging revealed pulmonary edema.

The patient was transferred to the intensive care unit for acute hypoxic respiratory failure. Infectious disease labs that were obtained on consultation at admission were positive for *Ehrlichia chaffeensis* by polymerase chain reaction. His antibiotics were narrowed to doxycycline, the patient clinically improved over the course of two weeks, and his labs normalized after a 10-day course of doxycycline.

## DISCUSSION

Human ehrlichiosis is a clinical syndrome characterized by fever and cytopenias. The median incubation period is eight days. Only 57% of confirmed cases are associated with a known tick bite.^4^ Fever is a prominent symptom (96%) along with malaise (77%), headache (72%), and myalgias (68%). Rash is seen uncommonly at presentation (6%), but approximately 26% of patients will develop a rash. Other symptoms include gastrointestinal symptoms (25 – 57%), cough (28%), and confusion (20%). The mortality rate is approximately 2%. Laboratory values can demonstrate an elevation in ALT and AST (84% of patients), leukopenia (61%), and thrombocytopenia (73%). Laboratory findings can include a mild-moderate elevation in alkaline phosphatase and LDH.^1^,^2^,^5^ Diagnosis is commonly achieved using PCR which is most sensitive during early infection. Paired indirect fluorescence antibody (IFA) assays are considered the gold-standard for serologic diagnosis, but take weeks to perform.^6^

Our patient’s course was longer than most and he had a period of brief improvement. He experienced approximately 20 days of relapsing fevers and worsening symptoms prior to hospitalization. A case series of ehrlichiosis as a cause of a prolonged fever found fevers ranged between 17 – 51 days.^7^ The patients in this series had various clinical manifestations, but defervescence occurred shortly after doxycycline therapy. One of these patients had the illness resolve after 30 days of a relapsing fever.

Case reports on whether ehrlichiosis is more severe in immunocompromised individuals are conflicting.^8^,^9^ A retrospective study by Thomas et al.^8^ found no significant difference in ICU admissions, duration of hospital stay, presenting lab values, or severity of illness between immune competent and immunosuppressed patients. Conversely, Safdar et al.^9^ found a 25% mortality rate for immune compromised individuals. In addition, they found that almost all 23 cases reviewed demonstrated significant organ dysfunction. Finally, a 2016 analysis of national surveillance data for *Ehrlichia chaffeensis* infections showed an increased risk of hospitalization, life-threatening complications, and death among immune compromised individuals. 3 This patient developed pulmonary edema and pleural effusions. Several case series found that pleural effusions or pulmonary involvement may be more common in the immunocompromised population manifesting as abnormal pulse oximetry, or abnormal pulmonary examination.^4^,^5^,^10^–^12^ A delay in diagnosis and by extension a delay in the administration of doxycycline, may explain the difference in severity. Delayed doxycycline therapy is associated with increased rates of ICU transfers, rates of mechanical ventilation, longer hospital stays, and overall length of illness.^13^

Lastly, filgrastim is associated with relatively common adverse effects of bone pain, fatigue, headache, and fever.^14^ His frequent injections with filgrastim early in the clinical course may have complicated the initial presentation by boosting his WBC count and, to some extent, his platelets and the non-specific adverse effects.

## CONCLUSION

Our patient’s diagnosis was nebulous secondary to a recent history of cytotoxic chemotherapy and immune modulating drugs. The similarity of the adverse effects of these medications with our patient’s clinical presentation of ehrlichiosis proved a diagnostic dilemma. The acute drop in an already low platelet count was an early suggestion of ehrlichiosis. In general, any patient presenting in the summer months with headache, fever, cytopenias, and/or elevated AST/ALT should be considered for empiric doxycycline therapy and diagnostic testing. In our review, we concluded that individuals with a compromised immune system develop more severe disease with *Ehrlichiosis spp*. It is unknown whether this is a product of individual susceptibility or delays in diagnosis. It is important to consider tick borne illnesses, including ehrlichiosis, in an immunocompromised patient presenting with fevers and cytopenias, especially if the patient has a recent history of a tick bite and lives in endemic area.
